# Diffusion Dialysis for Separation of Hydrochloric Acid, Iron and Zinc Ions from Highly Concentrated Pickling Solutions

**DOI:** 10.3390/membranes10060129

**Published:** 2020-06-24

**Authors:** Rosa Gueccia, Alba Ruiz Aguirre, Serena Randazzo, Andrea Cipollina, Giorgio Micale

**Affiliations:** 1 Department of Engineering, University of Palermo, viale delle Scienze Ed.6, 90128 Palermo, Italy; rosa.gueccia@unipa.it (R.G.); alba.ruizaguirre@unipa.it (A.R.A.); serena.randazzo@unipa.it (S.R.); giorgiod.maria.micale@unipa.it (G.M.); 2 CIEMAT PSA, Ctra. De Senés, 04200 Tabernas, Spain

**Keywords:** acid recovery, heavy metals, FeCl_2_, ZnCl_2_, industrial effluent treatment

## Abstract

Acid recovery from pickling waste solutions is an important step to enhance hot-dip-galvanizing industry process sustainability. Diffusion dialysis (DD) can be used to separate acids and heavy metals (e.g., iron and zinc) from pickling waters, promoting the circular use of such raw materials. In the present study, a laboratory scale unit operating in batch and a continuous large scale unit, both equipped with Fumasep anionic exchange membranes, were tested. Results obtained show that zinc and iron concentration affect the HCl recovery in opposite ways. Iron chlorides enhance acid recovery, while zinc chlorides considerably tend to diffuse through the membrane because of negatively charged chloro-complexes formation and slightly reduce the acid diffusion. A multi-components mathematical model, with a time-dependent and distributed-parameters architecture, was adopted enabling the prediction of operations with hydrochloric acid, zinc, and iron metals both in batch and in continuous dialyzers. As a result, a good comparison between model simulations and experiments was achieved in both configurations.

## 1. Introduction

Pickling is a key process in the hot-dip galvanizing industry. The most commonly used baths for carbon steel pickling contain hydrochloric acid solutions. Pickling with acids removes metallic and non-metallic substances from the steel surface assuring the surface quality required for subsequent treatment. Additionally, zinc-plated tools immersed in the pickling baths release some zinc ions in solution. As a result, acid is consumed and metals are dissolved in the bath in the form of iron and zinc chlorides reaching concentrations for the metal ions up to 200 and 10 g/L, respectively [1].

When a critical metals and acid concentration is reached, the pickling bath is considered spent. Therefore, from time to time, a portion of the pickling liquor has to be replaced. This stream has to be disposed and treated in centralized plants, often located hundreds or thousands kilometers away from the factory, thus representing a serious environmental and economic burden for the galvanizing company [2]. The specific waste composition depends on the plant and the pickling strategy adopted. Composition ranges of the pickling solutions are presented in Table 1, referring to the case of a hot-dip galvanizing plant situated in the south of Italy (Tecnozinco SrL, Carini, Italy). The lowest acid concentration and the highest metals concentrations are referred to the composition of pickling bath at the end of its life.

In addition to severe environmental and economic issues, the non-stable pickling conditions (due to the continuous consumption of acid and the increase in metals concentration) and the shutdown procedures for baths composition adjustments and cleanings/replacements can importantly affect the normal operation of the pickling process. Hence, the minimization of waste acid disposal and a look towards a cleaner production [3] are the major challenges for the sustainability of the process itself.

This topic has been widely addressed over the years and numerous solutions have been suggested in literature. Conventional treatment and neutralization technologies, such as alkaline neutralization and evaporation processes [1,4], are being substituted by innovative technologies either for the acid regeneration, such as the pyro-hydrolysis and the spray roasting [5,6], or for the acid recovery, such as ion exchange membrane technologies [7]. Interesting and more complex solutions for the selective recovery of the different valuable components dissolved in the spent pickling solution suggest the integration of different technologies [8,9].

Among these promising separation solutions, diffusion dialysis (DD) is compelling for its friendly environmental nature and process simplicity, reduced operating and investment costs, and low energy consumption [10,11]. No external driving force is required, separation between free acid, iron and zinc chlorides is promoted by a difference in concentration between two compartments separated by an anionic exchange membrane (AEM). Anion chlorides, for their negative nature, can diffuse through the AEM membrane, while bivalent metallic cations transport is denied by the positive fixed charges of the AEM membrane. Nevertheless, protons can cross the membrane due to their small dimension and the tunneling mechanism [12], accomplishing the electro-neutrality with chlorides and thus allowing for the acid recovery. 

On the other side, the co-existence of Zn^2+^ cations and Cl^−^ anions in the pickling solution, leads to the formation of negative chloro-complexes, which can considerably leak through the AEM, such as [ZnCl_3_]^−^ and [ZnCl_4_]^2−^ [13]. Several studies on inorganic acids coupled with a single metal salt are present in the literature [14,15,16]. Despite the loss of membrane permselectivity and transport properties in the presence of Zn ions have been addressed by different authors [17,18], concluding that HCl-ZnCl_2_ mixture separation exhibits low efficiency due to the strong attraction of Zn towards chlorides ions, the recovery of hydrochloric acid from waste acidic metal brines having real industrial composition in terms of Zn and Fe ions has not been investigate in detail. An experimental campaign aiming to investigate the effects of metal species on the recovery of inorganic acids was presented by Suk Jung Oh et al. [19], even though no modelling efforts were made to better characterize the system.

In this study, two DD modules equipped with Fumasep FAD-PET-75 anionic exchange membranes were used for assessing the separation of hydrochloric acid from highly concentrated zinc and iron solutions. A plate and frame laboratory-scale DD unit (10 × 10 cm^2^) operating in a batch configuration, presented in an authors’ previous work [20], was employed to extend the experimental investigation campaign considering also the effect of Zn ions in solution. In addition, a second large scale DD unit (10 × 80 cm^2^) operating in continuous mode was tested in order to present the operation of a real unit. A mathematical model, with a time and space distributed-parameters architecture, was further implemented in order to successfully simulate DD operations, also in the presence of a mixture of iron and zinc ions. The model was thoroughly validated with experimental data for the two configurations, highlighting its ability to well predict the observed experimental trends. The main outcomes of the DD modeling activities were implemented in a membrane processes integrated model for the design and development of a pilot scale unit for the waste acid recovery process [21].

## 2. Materials and Methods

### 2.1. Experimental

Experiments were operated with artificial solutions of 37% hydrochloric acid (Sigma Aldrich, St. Louis, MO, USA), iron chloride tetrahydrate (Sigma Aldrich, St. Louis, MO, USA ≥ 99%), zinc chloride (Carlo Erba, Val-de-Reuil, France ≥ 99%) and deionized water, prepared in laboratory. Sodium carbonate (Carlo Erba, Val-de-Reuil, France ≥ 99.5%) has been chosen as titrant and methyl orange solution 0.1% as indicator (Carlo Erba, Val-de-Reuil, France). 1,10-phenanthroline monohydrate (Sigma Aldrich, St. Louis, MO, USA, ≥ 99%), was used for iron detection in spectrophotometer analysis.

The retentate solutions, precisely concentrated feed solutions, were prepared by adding Fe ions with a concentration ranging from 50 to 150 g/L, Zn ions concentration from 5 to 20 g/L and HCl concentration from 70 to 100 g/L. The concentration ranges were opportunely chosen in order to make the laboratory artificial solutions comparable with the real industrial ones.

Whereas the diffusate solutions, streams in which acid is extracted, consist of deionized water, except for the tests carried out to explore the isolated zinc diffusive behavior through the membrane. In this respect, a solution of 3.6 g/L (0.1 mol/L) HCl was kept either in the retentate and diffusate solutions thus reducing acid and water fluxes thorough the membrane. 

The properties of the adopted Fumasep membrane (type FAD-PET-75, by Fumatech GmbH, Sankt Ingbert, Germany, https://www.fumatech.com/EN/Onlineshop/Products%2bof%2bHydrocarbon%2bpolymers/index.html) are reported in Table S1 in the supplementary information file.

### 2.2. Batch and Continuous Diffusion Dialysis Experimental Set-Up 

The experimental set-ups for the two operating configurations have similar characteristics. Both batch and continuous units consist of a plate and frame configuration with two endplates equipped with inlet-outlet manifolds, spacers (thickness 270 μm) and anion-exchange membranes interposed between the spacers. The solutions were fed to the DD units by two peristaltic pumps (Kronos, Lowell, MA, USA) placed right before the inlet manifolds, thus pressurizing both the retentate and diffusate channels. Two pressure gauges were installed in the circuit at the output of each pump in order to monitor the pressure drops inside the stack.

The detailed experimental set-up and procedures for the batch experiments are presented in Gueccia et al., 2019 [20]. An anion-exchange Fumasep FAD-PET-75 membrane (active area 10 × 10 cm^2^) was employed. Retentate and diffusate solutions were circulated from/to the same tanks thus realizing a batch operation mode. The pH and the conductivity were continually monitored by digital multi parameter pH/conductivity-meters (Hanna Instruments, Smithfield, WA, USA) in the two tanks and samples were withdrawn directly from the same recirculation tanks, the first one after 1 h, the others every 2 h. 

**Figure 1:**
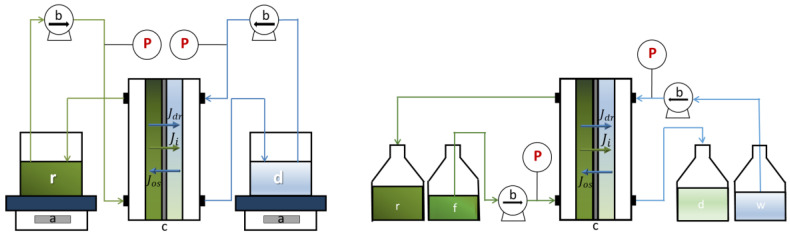
Sketch of the batch (on the left) and continuous (on the right) experimental systems. (a) balances, (b) peristaltic pumps, (c) Diffusion Dialysis units, (r) retentate buffer, (d) diffusate buffer, (f) feed tank, (w) deionized water tank, (P) pressure meter. The arrows denote the osmotic flux (*J_os_*), the drag flux (*J_dr_*) and the *i*-component flux (*J_i_*) across the membrane.

A sketch of the set-up is reported in Figure 1 (on the left side).

For the tests in continuous operating mode, 18 membranes (Fumasep FAD-PET-75 anionic exchange membrane, with active area of 80 cm length × 10 cm width) and 19 spacers with integrated gasket, thus leading to 9 feed and 10 diffusate channels, were placed in the large DD unit. Feed and deionized water solutions were fed to the DD unit in one-path countercurrent configuration, and they were drained out in the retentate and diffusate buffers, respectively.

Samples were collected in line at the outlet of the retentate and diffusate circuits. Steady state condition were assessed by monitoring the acid, iron and zinc concentration every 10 min in the samples. In all tests, steady state conditions were achieved after less than 20 min of operation (corresponding to about 5 times the residence time of solutions in the compartments).

A scheme of the continuous set-up is presented in Figure 1 (on the right side).

Both the batch and continuous DD equipment were assembled and provided by DEUKUM GmbH, Frickenhausen, Germany.

After the module assembly, a leakage test was executed to detect possible internal or external leakages by recirculating deionized water for 60 min. Before starting each experiment, membranes were conditioned in an acid and salts solutions for 120 min by fully filling the DD units with conditioning solutions. The conditioning solutions were prepared with an average concentration between the retentate and diffusate solutions for each specific test.

Error bars were obtained and showed in all the reported graphs by repeating each experiment at least two times.

Batch tests were run at ambient temperature (20–25 °C), by recirculating a set flow rate of 48 mL/min, corresponding to a channel linear velocity of 3 cm/s. Weight variations of the retentate and diffusate tanks were used for water flux determination.

By measuring volume (or total weight) and concentration variations, it is possible to characterize the system performances in terms of *i*-species fluxes ($J_{i}$), batch acid recovery ($RR_{HCl,t})\;$ and salt leakage ($Leakage_{salt,t})\;$ by means of simple transport and mass balance equations:

(1) $$J_{i}=-\frac{\Delta \left(V_{r}c_{i,r}\right)}{A_{m}\Delta t}=-\frac{{V_{r}|}_{t}}{A_{m}}\left(\frac{{c_{i,r}|}_{t+\Delta t}-{c_{i,r}|}_{t}}{\Delta t}\right)-\frac{{c_{i,r}|}_{t}}{A_{m}}\left(\frac{{V_{r}|}_{t+\Delta t}-{V_{r}|}_{t}}{\Delta t}\right)$$

(2) $$RR_{HCl,t}\left(\%\right)=\frac{{V_{d}c_{HCl,d}|}_{t+\Delta t}-{V_{d}c_{HCl,d}|}_{t}}{{V_{r}c_{HCl,r}|}_{t=0}}\times 100$$

(3) $$Leakage_{salt,t}\left(\%\right)=\frac{{V_{d}c_{salt,d}|}_{t+\Delta t}-{V_{d}c_{salt,d}|}_{t}}{{V_{r}c_{salt,r}|}_{t=0}}\times 100$$

with *V* being the tank volume, $c_{i}$ the bulk concentration of the *i*-component, $A_{m}$ the membrane area, and *t* the operation time. Subscripts *r* and *d* state for retentate and diffusate, respectively, while *t* denotes a variable being function of time.

Moreover, the acid recovery efficiency ($\eta_{HCl}$) parameter takes into consideration the ratio between actual and theoretical maximum acid recovery $\left(RR_{HCl}^{max}\right)$:

(4) $$\eta_{HCl}\left(\%\right)=\frac{RR_{HCl,t}}{RR_{HCl}^{max}}\times 100$$

More specifically the theoretical maximum recovery is defined as the highest acid recovery obtainable in equilibrium condition, which means 50% of the total initial acid amount when same volume for the retentate and diffusate tanks are considered.

A flow rate of 48 ml/min was adopted for the tests performed with the large-scale unit in continuous operating mode, corresponding to a channel linear velocity of 3 mm/s, typical industrial value for accomplishing a suitable components transport across the membranes. Continuous tests were run at ambient temperature (20–25 °C). For this configuration, the main two considered parameters, acid recovery ratio ($RR_{HCl}$) and iron and zinc leakage through the membrane ($Leakage_{salt}$) was evaluated in terms of flow rates as follow:

(5) $$RR_{HCl}\;\left(\%\right)=\frac{F_{d}c_{HCl,d}-F_{w}c_{HCl,w}}{F_{f}c_{HCl,f}}\times 100$$

(6) $$Leakage_{salt}\left(\%\right)=\frac{F_{d}c_{salt,d}-F_{w}c_{salt,w}}{F_{f}c_{salt,f}}\times 100$$

where *F* is the volumetric flow rate. Subscripts *f* and *w* state for feed and water, respectively.

### 2.3. Analysis

The acid concentration was detected by titration with Na_2_CO_3_ solutions using methyl orange as indicator. Iron ions concentration was revealed by spectrophotometry (spectrophotometer Beckham DU 800, Brea, CA, USA), by adding 1,10-phenanthroline. Water samples were characterized at a wave length of 510 nm. Zn detection was performed by atomic absorption (Shimadzu mod. AA6200, Kyoto, Japan).

## 3. Results and Discussion

### 3.1. Characterization of Zn Transport Behavior

A first experimental campaign with HCl and Zn was carried out through two different sets of tests in the batch experimental set-up. In the first one, an equal acid concentration of 3.6 g/L (0.1 mol/L) in the retentate and diffusate channels was used, while Zn ions were added in the retentate solution in two concentrations, 10 and 20 g/L, in order to isolate the behavior of zinc transport, from the retentate to the diffusate, across the membrane. Conversely, in the second set, HCl 73 g/L (2 mol/L) and different Zn ions concentrations (5, 10 and 20 g/L of Zn^2+^ ions) in the retentate and deionized water as inlet diffusate stream were used. 

**Figure 2:**
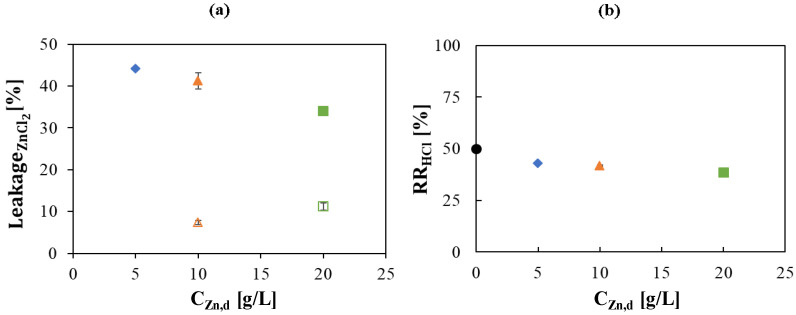
(**a**) ZnCl_2_ leakage percentage and (**b**) HCl Recovery Ratio vs. initial Zn concentration in the retentate: 0 (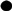) 5 (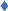), 10 (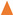) and 20 g/L (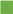). Initial acid concentration: 3.6 g/L (0.1 mol/L) (empty symbols) and 73 g/L (2 mol/L) (solid symbols). Solutions flow rate fixed at 48 mL/min. Feed solution: HCl + ZnCl_2_. Inlet diffusate: 3.6 g/L (0.1 mol/L) HCl solution (empty symbols) and deionized water (solid symbol). Batch experimental-set-up.

The main outcome obtained from the comparison between these experiments is a strong influence of HCl concentration on Zn membrane permeability. As reported in Figure 2a, Zn concentration increase is expressed in a Zn leakage growth through the membrane from 7 to 11%, when HCl concentration is comparable in the two compartments, as clearly the Zn transport driving force increases with concentration. Higher leakages and a counter trend are observed when feed acid concentration is 73 g/L (2 mol/L), as a leakage reduction from 41 to 34% is observed. This can be explained considering that the common Cl^−^ ion from the acid molecules in solution promotes zinc tendency to form negatively-charged chloro-complexes, which are more permeable through the anionic membrane, hence explaining the higher leakage values. Our results are in agreement with those published by other authors [16], where the transport properties of a Neosepta- AFN anion-exchange membrane in contact with aqueous solutions containing zinc chloride at different acid and zinc concentrations were investigated.

The decreasing trend of the Zn leakage with metal concentration increase is due to the reduced relative possibility of zinc chloro-complexes formation, since the initial acid concentration is invariable. 

HCl recovery is influenced, consequentially, as demonstrated in Figure 2b. The acid recovery with zinc in solution is lower than the value without the zinc metal in solution, with an asymptotic tendency to a value slightly lower than 40%. 

Thus, an acid recovery efficiency $\eta_{HCl}\;$ lower than 100% was observed for all the tests, contrarily to the case of HCl-FeCl_2_ mixture [15, 20]. Indeed, Luo et al. [10] demonstrated that the Cl^−^ permeability coefficient for the HCl-ZnCl_2_ system is the lowest among all the other investigated systems, which is in agreement with the above finding.

**Figure 3:**
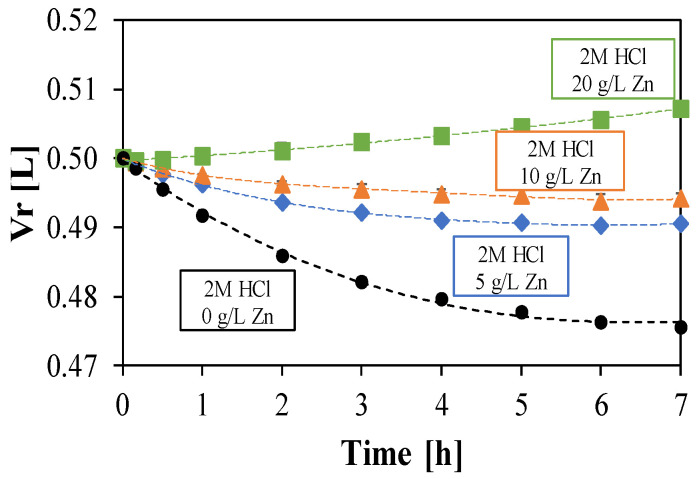
Trend over time of the retentate tank volume. Initial Zn conc.: 0 (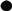); 5 (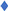), 10 (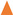) and 20 (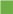) g/L. Initial HCl concentration: 73 g/L (2 mol/L). Solutions flow rate fixed at 48 mL/min. Feed solution: HCl + ZnCl_2_. Inlet diffusate: deionized water. Batch experimental-set-up.

Figure 3 shows the volume variation in the feed tank against the test duration, which is due to the net water flux through the membrane. 

Concentration difference between the two channels generates an osmotic pressure resulting in water passage from the diluted to the concentrated compartment. However, an opposite water flux is realized as result of the water molecules dragging for hydration of the acid and metals molecules. The acting directions of the two fluxes are schematized in Figure 1.

A reversal trend within the examined concentration range is observed: the feed volume is reduced over the time at lower zinc concentrations (0, 5, and 10 g/L), resulting in a prevailing drag flux, whereas an opposite behavior characterizes the volume variation at the higher concentration (20 g/L), where the osmotic pressure is enhanced due to the higher salt concentration difference between the feed and the retentate compartment.

### 3.2. HCl, Fe and Zn Test: Mutual Effects

The combined effects of the main ions present in a pickling solution were considered in order to better predict the behavior in a real environment. Artificial solutions with Fe, Zn, and HCl in the range of typical industrial pickling solutions were prepared and tested in laboratory. In this respect, four different tests were carried out by alternatively changing the concentration of a single metal and keeping the HCl concentration at 73 g/L (2 mol/L). In particular, the iron concentration was varied from 100 to 150 g/L, while the zinc concentration from 5 to 10 g/L.

**Figure 4:**
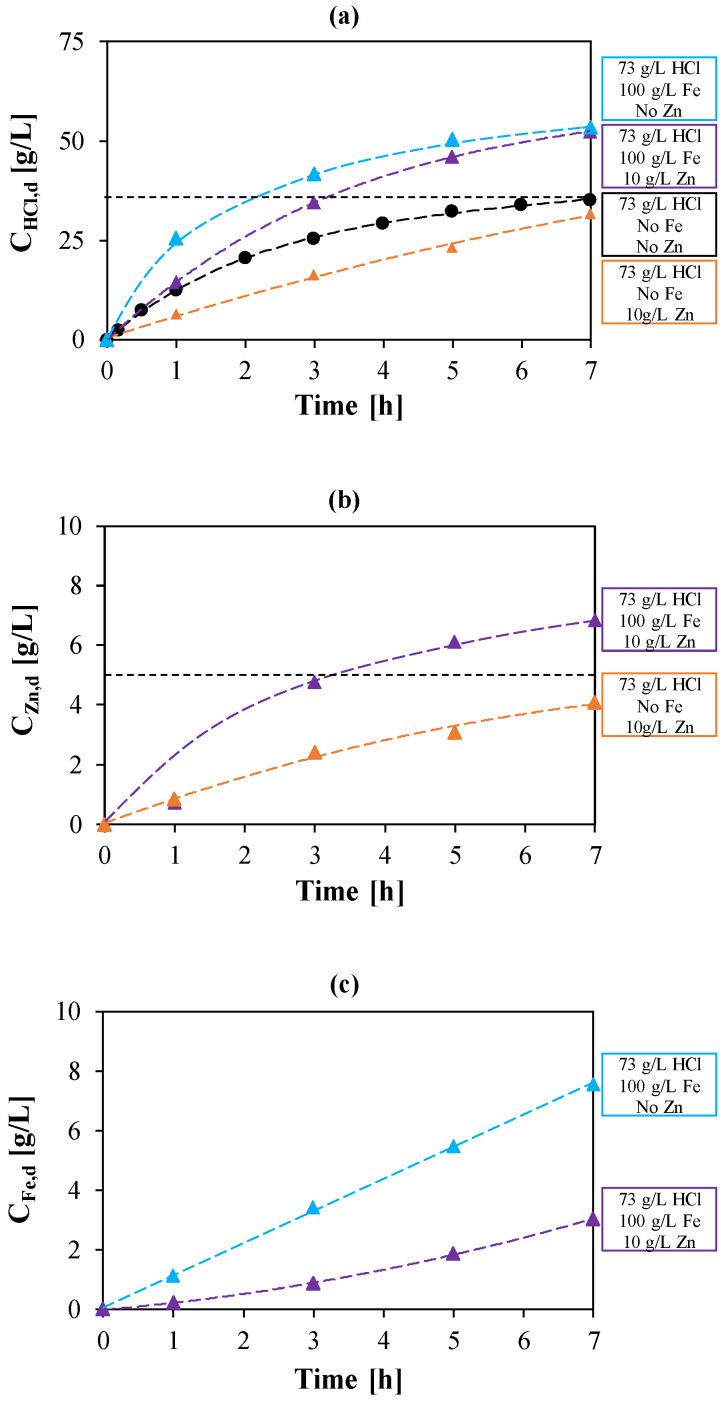
Temporal trend of HCl concentration (**a**), Zinc concentration (**b**) and Iron concentration (**c**) in the diffusate. Initial Zn concentrations: 0 (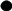,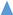) and 10 (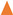,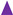) g/L. Initial Fe conc.: 0 (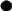,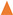); 100 (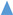,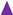) g/L. Initial acid conc.: 73 g/L (2 mol/L). Solutions flow rate fixed at 48 mL/min. Feed solution: HCl + FeCl_2_ + ZnCl_2_. Inlet diffusate: deionized water. Batch experimental-set-up.

Experimental results for the test with initial Fe and Zn concentrations of 100 and 10 g/L, respectively, were considered as reference case to describe and characterize all the phenomena involved in HCl, Fe and Zn mixtures in comparison with the solutions of HCl coupled with a single salt. The concentration profiles for HCl, Fe, and Zn are reported versus time in Figure 4.

As reported in Figure 4a, HCl flux is reduced by the presence of Zn due to the competitive flux of the negative Zinc-chloro complexes, as already mentioned. Nevertheless, in the tests with Fe, the considerable amount of iron chlorides in solution causes a supplementary driving force for the diffusion of acid molecules (salt effect), resulting in acid recovery efficiencies over 100% and, in more detail, 116% and 125% for the test with the three components and the HCl + Fe test, respectively. This result shows the crucial behavior of iron salt to achieve high acid recoveries. 

The effect of iron chloride on Zn permeation can be reasonably expected: it supplies additional chlorides ions for the zinc chloro-complexes formation, as previously discussed for the acid, resulting in a higher flux through the membrane. Indeed, the zinc concentration in the diffusate is much higher when iron is also present in the feed solution (Figure 4b).

On the other side, the leakage of iron through the membrane undergoes an important decrease due to the competitive passage of Zn, as shown in Figure 4c.

Similar considerations can be derived for tests run with the continuous dialyzer. Continuous operation tests were performed with different feed composition in order to validate the reproducibility of all the effects described for the batch tests. In Table 2, all the tests performed with the continuous dialyzer are reported.

Results obtained are in agreement with previously presented effects. 

It is worth noting that for better assessing results reliability, the tests n. 2 and 4 were repeated three times and the experimental error has been estimated and reported in Table 3.

Table 3 reports the value of the main performance parameters for all the continuous operation tests.

The test n.4, with 100 g/L HCl, 117 g/L of Fe and 8 g/L of Zn in the feed stream and deionized water as diffusate stream, the most relevant one as it covers all phenomena under investigation, is commented in details. The particular composition chosen for the feed solution is the designed operating composition at which the pickling bath would efficiently work in view of the continuous regeneration.

Concerning the acid recovery, despite the reduction of acid flux due to the competitive presence of zinc, the acid recovery settles around a relatively high value of 79%. A similar acid recovery was reported in Oh et al. [19], although in their study the investigated iron and zinc concentration range was considerably lower (5–30 g/L and 2–3 g/L, respectively), and the acid concentration higher (up to 180 g/L). This confirms the crucial importance of the iron ions in the recovery efficiency, as a comparable acid recovery rate is gained at lower HCl and higher Zn concentrations. The iron leakage through the membrane (about 30%) is slightly lower than the tests n.3 (with only HCl and FeCl_2_), but much lower compared to the zinc one (60%). However, it reaches significantly higher values than in batch operations, likely due to the higher residence time of solutions in feed and diffusate compartments. The membrane, as expected, is highly permeable to zinc. The high initial concentration in HCl and FeCl_2_ is expressed in high concentration of Cl^−^ ions, which are available for the zinc chloro-complex formation. However, the initial amount of zinc is much lower than the iron one, thus the concentration in the diffusate stream can be acceptable in the view of reusing it as recovered pickling solution. Indeed, the concentration of iron in the diffusate stream reaches values up to 65 g/L while the zinc concentration is about 9 g/L. Concentration values for the test n.4 are reported in the next section.

## 4. Modelling Diffusion Dialysis with Multi-Metals Solutions

A modelling tool, already presented in a previous work [20], was further implemented and adapted so as to predict the DD process under steady state and transitory functioning when feeding acid solutions with multi-metal ions mixtures. 

The DD model has a 1-dimensional distributed parameters spatial discretization along the channel length dimension with steady-state spatial differential mass balance equations, thus providing information on how concentrations vary along the channel and allowing the simulation of co- and counter-current system configurations. 

The outputs of the DD model, namely concentrations and flow rates of both streams, are used as inputs in a dynamic model for the entire test-rig. The dynamic model consists of time-differential equations with a time discretization in order to simulate the time-dependent variation of ions concentrations and volumes in the solutions buffers, when the system is operated in batch. The detailed structure of the above mentioned model is described in depth in Gueccia et al. [20], where an algorithm for the numerical implementation of the spatial-time dependent model is reported, showing how the two different model sections are linked together.

The model has also been adapted to simulate the larger DD unit, by wider discretization along the channel length and accounting for the *n* number of feed/diffusate channels in one unit.

### 4.1. Modification in Model Constitutive Equations 

Osmotic pressure evaluation has been improved by including in the model structure the Pitzer model equations instead of the Van´t Hoff correlations adopted in Gueccia et al. [20]. As a result, the osmotic pressure [22] has been evaluated as reported below:

(7) $$\pi =\frac{RTM_{s}\phi }{1000v_{s}}\cdot \sum^{}{\left(im\right)}_{i}$$

where *R* is the ideal gases constant of, *M_S_* is the molecular weight of the solvent, *v_s_* is the solvent molar volume, *i* is the Van´t Hoff coefficient of the i-component, *m_i_* is the molal concentration of the i-component, and *π* is the osmotic pressure. The osmotic coefficient (ϕ) was calculated according to Pitzer equations [23]. Hydrochloric acid, iron, and zinc chlorides Pitzer parameters were extrapolated from the literature [24,25]. 

In order to properly implement the above Equation (7), dissociation reaction equilibria were studied (details on equilibrium equations are reported in the materialthe in the Supplementary Materials in Table S2). In particular, at the pH of the experiments (typically, *pH* < 1), for single iron chlorides solutions, iron is present as a mixture of Fe^2+^ (30%) and FeCl^+^ (70%), while for single zinc chlorides solutions, the salt is present as a mixture of Zn^2+^ (15%), ZnCl^+^ (14%), ZnCl_2aq_ (15%), ZnCl_3_^−^ (30%), ZnCl_4_^2−^ (26%). Therefore, over 50% of zinc is present as negatively-charged complex, thus strengthening the argument of high zinc leakage through the membrane.

The hydrochloric acid, for its strong acidic strength, is totally dissociated in water solution. Corresponding to this, the Van´t Hoff coefficient for HCl was 2, for FeCl_2_ was 2.3 and for ZnCl_2_ was 1.6.

### 4.2. Model Calibration

Calibration of permeability coefficients was performed in order to consider also the presence of zinc species, by identifying possible dependences with its concentration in the feed solution.

In particular, all transport equations were re-adapted including correction terms, which account for the effect of co-ions present in solution promoting or hindering the passage of species as already phenomenological described in Section 3 and reported by some authors [15,26].

Firstly, the adapted model was applied to derive the Zn permeability ($P_{ZnCl_{2}}$) through the membrane by considering the experimental data with HCl 3.6 g/L (0.1M) in the two compartments. Then, the case of retentate solution composed by a mixture of HCl and ZnCl_2_ was considered in order to find the additional zinc permeability related to the additional chloride provided from the hydrochloric acid $(U_{ZnCl_{2}}^{HCl}$). At the same time, the detrimental effect on the acid flux was characterized by a secondary correction factor $\left(U_{HCl}^{ZnCl_{2}}\right)$. In particular, the transport equations for HCl and Zn solutions are reported below:

(8) $$J_{ZnCl_{2}}=U_{ZnCl_{2}}\left(c_{ZnCl_{2},r}-c_{ZnCl_{2},d}\right)+U_{ZnCl_{2}}^{HCl}\left(c_{HCl,r}-c_{HCl,d}\right)$$

(9) $$J_{HCl}=U_{HCl}\left(c_{HCl,r}-c_{HCl,d}\right)-U_{HCl}^{ZnCl_{2}}\left(c_{ZnCl_{2},r}-c_{ZnCl_{2},d}\right)$$

where $U_{ZnCl_{2}}$ and $U_{HCl}\;$ are the overall mass transfer coefficients of the zinc and acid component, respectively.

**Figure 5:**
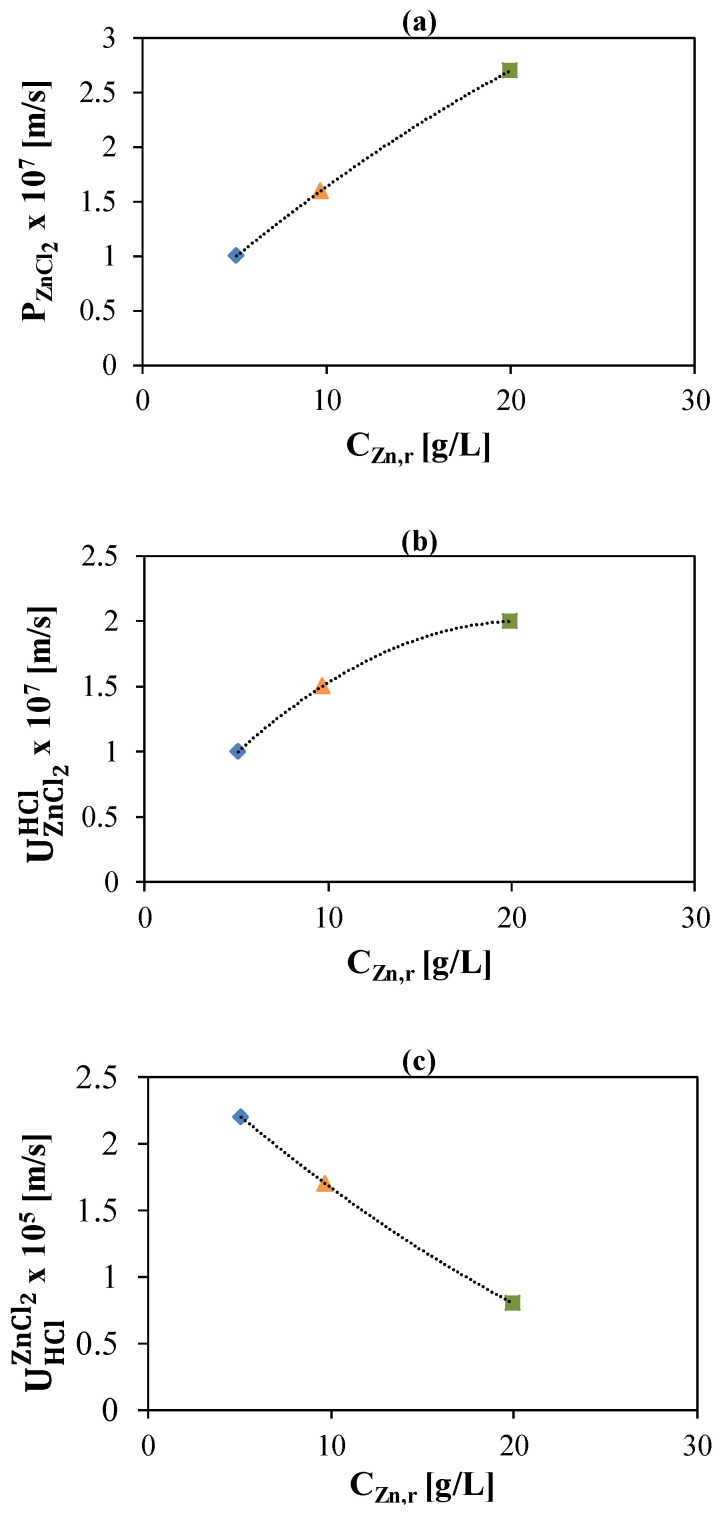
Model calibration results in terms of variation of the zinc diffusive permeability (**a**)**,** additional Zn mass coefficient (**b**) and additional HCl mass coefficient (**c**) as a function of Zn concentration in the feed compartment.

As graphically shown in Figure 5, constant values for each test were considered (three different points) and a reasonable agreement between model predictions and experiments was observed when using linear correlations linking the acid (*P_HCl_*) permeability and the water osmotic permeability (*P_os_*) to the concentration, as derived in a previous work [20]. Therefore, the constant parameters have been effectively correlated with the zinc concentration using simple quadratic equations, as reported below:

(10) $$P_{ZnCl_{2}}=-2.7\times 10^{-6}c_{r,ZnCl_{2}}^{2}+1.7\times 10^{-6}c_{r,ZnCl_{2}}+0.7\times 10^{-8}$$

(11) $$U_{HCl}^{ZnCl_{2}}=6.1\times 10^{-5}c_{r,ZnCl_{2}}^{2}-8.5\times 10^{-5}c_{r,ZnCl_{2}}+2.8\times 10^{-5}$$

(12) $$U_{ZnCl_{2}}^{HCl}=-1.7\times 10^{-6}c_{r,ZnCl_{2}}^{2}+1.1\times 10^{-6}c_{r,ZnCl_{2}}+0.2\times 10^{-7}$$

where $U_{HCl}^{ZnCl_{2}}$ accounts for the HCl flux reduction related to the Zn presence; $U_{ZnCl_{2}}^{HCl}$ is the additional mass coefficient for the Zn passage due to the HCl concentration difference between the two compartments.

Although most trends shown in Figure 5 are almost linear, the choice of 2nd order correlations resulted in a more accurate fitting, thus being the final choice for the model.

The zinc diffusive permeability, as reported in Figure 5a, increases as Zn concentration increases in the entire examined span. This value is 10 times lower than the acid permeability [20].

Likewise, the additional mass coefficient for the Zn passage is an empirical law gained from experiments comparison which shows an increasing trend with the zinc concentration rising (Figure 5b). Despite the lower zinc permeability value, this last contribution acts increasing significantly the zinc losses through the membrane.

Conversely, for the acid reduction mass coefficient, a decreasing trend is observed with the zinc concentration increases, as shown in Figure 5c. The acid flux reduction is related to the zinc chloro-complexes formation, which is less promoted at higher zinc concentrations.

In addition, the osmotic permeability coefficient $\left(P_{os}\right)$ was adjusted as result of the Pitzer model implementation for calculating osmotic pressure, leading to the following new correlation:

(13) $$P_{os}=1.3\times 10^{-7}c_{r,HCl}^{2}-9\times 10^{-7}c_{r,HCl}+8.1\times 10^{-6}$$

When industrial solutions are reproduced in laboratory, by merging all the components with their common concentrations, also the mutual effect between the two metals it is relevant to be figured out. The iron salt enhances the zinc passage for the Cl^−^ common ion, providing a further contribution to the Zn mass diffusion through the membranes $\left(U_{ZnCl_{2}}^{FeCl_{2}}\right)$:

(14) $$U_{ZnCl_{2}}^{FeCl_{2}}=1.5\times 10^{-6}c_{r,ZnCl_{2}}^{2}+3.9\times 10^{-7}c_{r,ZnCl_{2}}+6.2\times 10^{-8}$$

In the other hand, the iron flux is competitively reduced by the zinc flux, and the rate of this reduction was experimentally estimated to be of 30% of the iron flux itself.

Therefore, it is possible to evaluate the flux laws of the three different components [27] across the membrane, also considering the acid and iron permeability values obtained in the previous work [20].

(15) $$J_{HCl}^{tot}=U_{HCl}\left(c_{HCl,r}-c_{HCl,d}\right)+U_{HCl}^{FeCl_{2}}\left(c_{FeCl_{2},r}-c_{FeCl_{2},d}\right)-U_{HCl}^{ZnCl_{2}}\left(c_{ZnCl_{2},r}-c_{ZnCl_{2},d}\right)$$

(16) $$J_{FeCl_{2}}^{tot}=0.7U_{FeCl_{2}}\left(c_{FeCl_{2},r}-c_{FeCl_{2},d}\right)$$

(17) $$J_{ZnCl_{2}}^{tot}=U_{ZnCl_{2}}\left(c_{ZnCl_{2},r}-c_{ZnCl_{2},d}\right)+U_{ZnCl_{2}}^{HCl}\left(c_{HCl,r}-c_{HCl,d}\right)+U_{ZnCl_{2}}^{FeCl_{2}}\left(c_{FeCl_{2},r}-c_{FeCl_{2},d}\right)$$

where $U_{HCl}^{FeCl_{2}}$ accounts for the HCl flux increase related to the iron effect; $U_{FeCl_{2}}$ is the overall mass transfer coefficient of the iron component.

### 4.3. Model Validation

**Figure 6:**
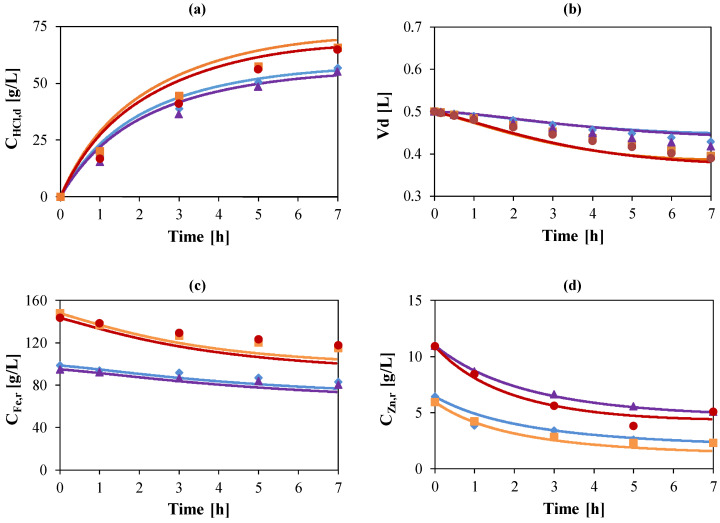
HCl concentration in diffusate (**a**), diffusate volume (**b**), Fe concentration in retentate (**c**) and Zn concentration in retentate (**d**) vs. time. Initial Zn concentrations: 5 (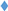,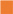) and 10 (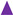,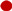) g/L. Initial Fe concentrations: 100 (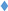,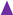) and 150 (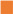,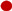) g/L. Initial acid concentrations: 73 g/L (2 mol/L). Flow rate: 48 mL/min. Feed: HCl, FeCl_2_, ZnCl_2_ solution. Diffusate IN: deionized water. Theoretical curves (—) obtained by using the model.

As result of this experimental campaign, exhaustive correlations both for the single components permeability and for their mutual influences were obtained. Model validation was achieved by comparing simulation outcomes with the experiments. This is reported in Figure 6, which compares experimental and simulated trends when the typical industrial compositions are considered.

**Figure 7:**
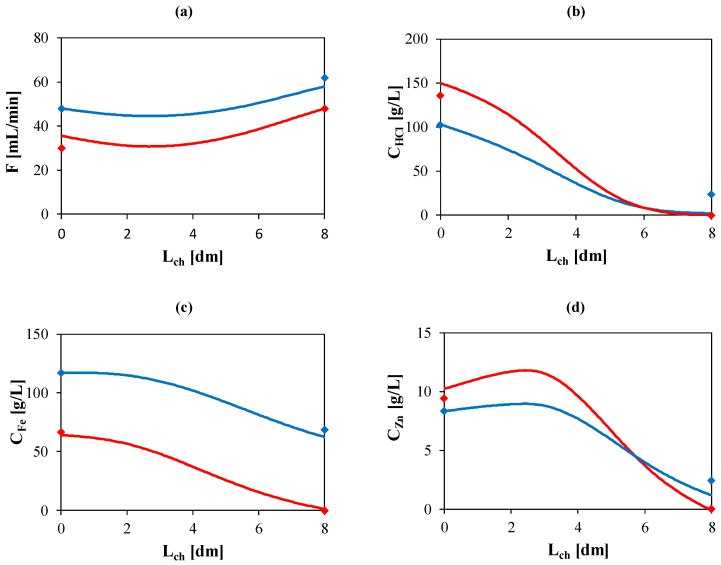
Flow rate (**a**), HCl concentration (**b**), Fe concentration (**c**) and Zn concentration (**d**) vs. channel length for retentate (blue) and diffusate (red) for the continuous test 4 named in Table 2. Theoretical curves (—) obtained by using the model. Experimental data (dots).

As a further validation of the proposed model, the correlations obtained were used to predict the operation of the large-scale unit. The predicted concentration and flow rates along the channel are shown in Figure 7, where they are compared with the inlet and outlet experimental measurements. 

Of interest, the acid diffusate concentration at the outlet of the unit is larger than the feed concentration thanks to the salt effect, though the osmotic flux, reflecting in a reduction of the diffusate flow rate along the channel, restricts the RR below 80%. Same behavior is observed for the Zn concentration profile, even though the initial low amount of the component let to have a low concentration in the recovered acid stream. A lower passage of iron is observed in agreement with the batch results. The discrepancy between the experimental data and the predicted values can be ascribed to the expected fluxes reduction when the net driven force approaches to values close to zero. The suggested correlations do not consider these phenomena and overestimate the fluxes in these ranges.

**Figure 8:**
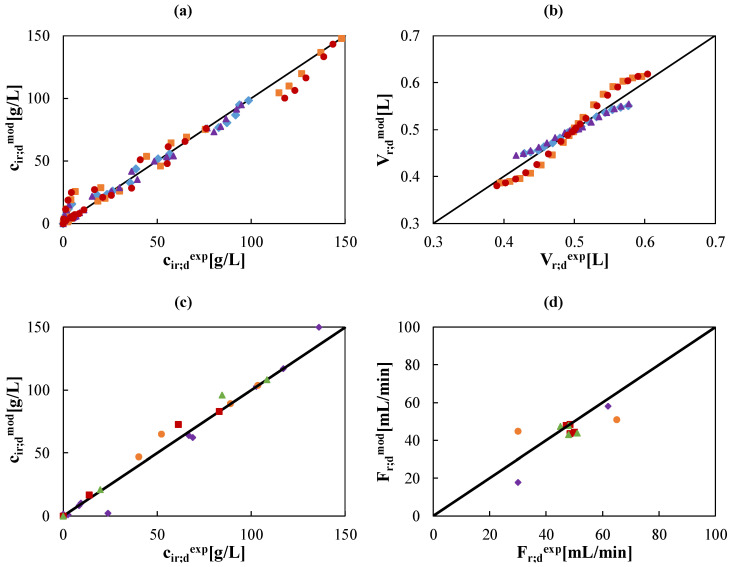
Parity plots of all experimental (exp) and predicted (mod) values. (**a**)&(**b**) species concentration and tanks volumes in retentate and diffusate compartments for tests with initial HCl at 73 g/L (2 mol/L), Zn at 5 (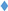,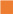) and 10 (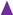,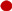) g/L, and Fe at 100 (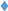,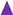) and 150 (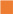,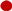) g/L. (**c**)&(**d**) species concentration and flow rates in retentate and diffusate streams for tests 1 (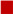) 2 (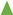), 3 (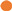), and 4 (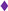) named in Table 2.

Finally, a comprehensive validation of the DD model, when the three components are included in solution, is presented in the form of parity plots in Figure 8, where all experimental data within the batch and continuous configurations are displayed against model prediction data.

## 5. Conclusions

Hydrochloric acid recovery from highly concentrated iron and zinc chlorides solutions by diffusion dialysis (DD) was explored.

Two different DD units (a laboratory- and a large-scale), equipped with Fumasep FAD anionic exchange membranes, were used for characterizing the behavior of the system fed by artificial solution mimicking the real industrial solutions composition. The main effects of operating parameters were highlighted and presented as process performance variations.

At first, isolated influence of zinc salt on the acid recovery was explored. The HCl recovery was reduced from 50% (maximum value for a batch configuration) to 40%, caused by the presence of negative zinc-chloro complexes that easily diffuse through the membrane.

Then, the combined effect of iron and zinc chlorides on the separation of HCl was also considered. For the flux of acid, deviation from the pure acid solution behavior can be correlated to two competitive phenomena: the enhancing iron effect and the competitive zinc flux. As a result, in batch tests high acid recovery efficiency values were gained (recovering all the acid until equal concentration in the retentate and diffusate are reached, or even more in some cases), thus denoting the fundamental and synergic influence of iron effect in the HCl recovery achievement. 

Zinc leakage through the membrane firmly depends on the presence of both HCl and FeCl_2_, which has led to an increase of the zinc leakage across the membrane.

All such findings have been found entirely in agreement with the results obtained with the continuous large-scale unit. Despite the reduction of acid flux due to the presence of zinc, up to 80% of the free acid has been recovered. Zinc leakage can be twice than the iron one. However, because of the lower zinc concentration, with respect to the other components, it is possible to reuse the diffusate solution in the pickling process.

Both continuous and batch operations were simulated by the implementation of a time-dependent and space distributed-parameters multicomponent model. The model has been calibrated and fully validated with relevant data collected during the wide experimental campaign. This valid tool can be applied for the elaboration of further routes for system optimization.

## Figures and Tables

**Table 1:** Acid and metals concentration ranges of a pickling solution (data provided from Tecnozinco SrL, Carini, Italy).

Component	Unit	Mean
Free acidity (HCl)	g/L	20–150
Fe	g/L	50–150
Zn	g/L	1–20

**Table 2:** Summary of tests performed with the continuous dialyzer. Flow rate refers to both diffusate and dialysate. Concentration values reported indicate the initial feed concentration for the different species. Diffusate solution consists of deionized water.

№ Test Identification	№ of Repetitions	Flowrate mL/min	*C*_HCl,f_ g/L	*C*_Fe,f_ g/L	*C*_Zn,f_ g/L
1	1	48	73	-	-
2	3		100	-	-
3	1		100	117	-
4	3		100	117	8

**Table 3:** Acid recovery and salt leakages for continuous operation tests. Values between brackets indicate the standard deviation.

№ Test Identification	${RR}_{HCl}\left(\%\right)$	$Leakage_{FeCl_{2}}\left(\%\right)$	$Leakage_{ZnCl_{2}}\left(\%\right)$
1	76	-	-
2	80 (± 1.8%)	-	-
3	87	35	-
4	79 (± 6.0%)	30 (± 7.8%)	60 (± 7.7%)

## Supplementary Materials

Supplementary materials can be found at https://www.mdpi.com/2077-0375/10/6/129/s1. Table S1: Properties of Fumasep FAD type anion exchange membrane. Table S2: Dissociation reactions for the HCl+ ZnCl2 and HCl+ FeCl2 systems and relative equilibrium constants (source: PHREEQC Dissociation).

## Nomenclature and Abbreviations

DD	-	diffusion dialysis
A	[$m^{2}$]	area
c	$\left[\frac{\mathrm{mol}}{l}\right]$	molar concentration
F	$\left[\frac{l}{s}\right]$	volumetric flow rate
FeCl_2_	[-]	iron chloride (II)
HCl	[-]	hydrochloric acid
$J_{i}$	$\left[\frac{\mathrm{mol}}{m^{2}\cdot s}\right]$	molar flux
$J_{w};J_{os};J_{dr}$	$\left[\frac{l}{m^{2}\cdot s}\right]$	volumetric flux
i	[-]	Van´t Hoff coefficient
IN	[-]	initial
m	$\left[\frac{\mathrm{mol}}{\mathrm{kg}}\right]$	molality
M	$\left[\frac{g}{\mathrm{mol}}\right]$	molecular weight
$P_{i}$	$\left[\frac{m}{s}\right]$	diffusive permeability
$P_{os}$	$\left[\frac{l}{\mathrm{bar}\cdot m^{2}\cdot s}\right]$	osmotic permeability
R	$\left[\frac{l\cdot \mathrm{bar}}{K\cdot \mathrm{mol}}\right]$	gas constant
RR	[%]	recovery ratio
T	[K]	temperature
U	$\left[\frac{m}{s}\right]$	overall mass transfer coefficient
V	[l]	volume
v	$\left[\frac{m}{s}\right]$	linear velocity
ZnCl_2_	[-]	zinc chloride
β	[-]	hydration number
η	[%]	recovery efficiency
v	$\left[\frac{l}{\mathrm{mol}}\right]$	molar volume
π	[bar]	osmotic pressure
ϕ	[-]	osmotic coefficient
**Subscripts and Superscripts**		
ch	channel	
d	diffusate	
dr	drag	
exp	experimental	
f	feed	
i	ith component (HCl or FeCl_2_ or ZnCl_2_)	
m	membrane	
max	maximum	
mod	modelling	
os	osmotic	
r	retentate	
s	solvent	
t	time	
tot	total	
w	water	
