# Supplementary material for: Diffusion Dialysis for Separation of Hydrochloric Acid, Iron and Zinc Ions from Highly Concentrated Pickling Solutions
Source: Membranes (Basel). 2020 Jun 24;10(6):129. doi: 10.3390/membranes10060129 (PMC7344563; doi:10.3390/membranes10060129)
Supplement: Supplementary file 1 [file membranes-10-00129-s001.pdf]

membranes

Supplementary Materials: Diffusion Dialysis for Separation of Hydrochloric Acid, Iron and Zinc Ions from Highly Concentrated Pickling Solutions

Rosa Gueccia [1], Alba Ruiz Aguirre [1,2], Serena Randazzo [1], Andrea Cipollina [1,*], Giorgio Micale [1]

[1] Department of Engineering, University of Palermo, viale delle Scienze Ed.6, 90128 Palermo, Italy; rosa.gueccia@unipa.it (R.G.); alba.ruizaguirre@unipa.it (A.R.A.); serena.randazzo@unipa.it (S.R.); giorgiod.maria.micale@unipa.it (G.M.)

[2] CIEMAT PSA, Ctra. De Senés, 04200 Tabernas, Spain

* Correspondence: andrea.cipollina@unipa.it

1 Membrane Properties

The properties of the Fumasep membranes (type FAD-PET-75, by Fumatech GmbH, https://www.fumatech.com/EN/Onlineshop/Products%2bof%2bHydrocarbon%2bpolymers/index.html) are presented in Table S1.

Table S1. Properties of Fumasep FAD type anion exchange membrane.

Item	Unit	Specifications
thickness (dry)	µm	70-80
electric resistance [a)]	Ω cm^2	<1
selectivity [b)]	%	>90
stability	pH	<9
ion exchange capacity	meq/g	>1.5
specific conductance [c)]	mS/cm	> 13
weight per unit area	mg/cm^2	9-12
proton (H^+) transfer rate [d)]	$mmol/min/cm^2$	>1500
Young's modulus [e)]	MPa	>1000
tensile strength [e)]	MPa	>40
elongation at break [e)]	%	>15
bubble point test in water at T = 25 °C	bar	>3

[a)] in Cl^- form in 0.5 M NaCl at T = 25°C, measured in standard measuring cell (through-plane)

[b)] determined from membrane potential measurement in a concentration cell 0.1/0.5 M KCl at T = 25°C

[c)] determined in Cl^- form in 0.5 NaCl at T =30°C

[d)] determined from pH potential measurement in a concentration cell 0.5 M HCl/0.5 M KCl at T = 25°C

[e)] determined by stress-strain measurement at T = 25°C and 50 % r.h., according to DIN EN 527-1

2. Equilibrium Species Dissociation

Dissociation reactions equilibria for the HCl+ $ZnCl_2$ and HCl+ $FeCl_2$ systems are reported in Table S2 where also the equilibrium dissociation constants are shown (source: PHREEQC software, https://www.usgs.gov/software/phreeqc-version-3).

Table S2. Dissociation reactions for the HCl+ $ZnCl_2$ and HCl+ $FeCl_2$ systems and relative equilibrium constants (source: PHREEQC software).

Dissociation reaction	$-\log K_{eq}$	Equilibrium Constant K_{eq}
$ZnCl^+ \leftrightarrow Zn^{2+} + Cl^-$	0.43	$k_1 = 0.37$
$ZnCl_2 \leftrightarrow Zn^{2+} + 2Cl^-$	0.45	$k_2 = 0.35$
$ZnCl_3^- \leftrightarrow Zn^{2+} + 3Cl^-$	0.5	$k_3 = 0.32$
$ZnCl_4^{2-} \leftrightarrow Zn^{2+} + 4Cl^-$	0.2	$k_4 = 0.63$
$FeCl^+ \leftrightarrow Fe^{2+} + Cl^-$	0.43	$k_1 = 0.37$

Different species concentration for the HCl+ $ZnCl_2$ system were assessed from Equations (S1) to (S7):

$$[ZnCl^+] = \frac{[Zn^{2+}][Cl^-]}{k_1}\frac{\Upsilon_{Zn^{2+}}\Upsilon_{Cl^-}}{\Upsilon_{ZnCl^+}} \quad \text{(S1)}$$

$$[ZnCl_2] = \frac{[Zn^{2+}][Cl^-]^2}{k_2}\frac{\Upsilon_{Zn^{2+}}\Upsilon_{Cl^-}^2}{\Upsilon_{ZnCl_2}} \quad \text{(S2)}$$

$$[ZnCl_3^-] = \frac{[Zn^{2+}][Cl^-]^3}{k_3}\frac{\Upsilon_{Zn^{2+}}\Upsilon_{Cl^-}^3}{\Upsilon_{ZnCl_3^-}} \quad \text{(S3)}$$

$$[ZnCl_4^{2-}] = \frac{[Zn^{2+}][Cl^-]^4}{k_4}\frac{\Upsilon_{Zn^{2+}}\Upsilon_{Cl^-}^4}{\Upsilon_{ZnCl_4^{2-}}} \quad \text{(S4)}$$

$$[Zn]_{TOT} = [ZnCl^+] + [ZnCl_2] + [ZnCl_3^-] + [ZnCl_4^{2-}] + [Zn^{2+}] \quad \text{(S5)}$$

$$[Zn^{2+}] = \frac{[Zn]_{TOT}}{1 + [Cl^-]\Upsilon_{Zn^{2+}}\Upsilon_{Cl^-}\left(\frac{1}{k_1}\frac{1}{\Upsilon_{ZnCl^+}} + \frac{[Cl^-]}{k_2}\frac{\Upsilon_{Cl^-}}{\Upsilon_{ZnCl_2}} + \frac{[Cl^-]^2}{k_3}\frac{\Upsilon_{Cl^-}^2}{\Upsilon_{ZnCl_3^-}} + \frac{[Cl^-]^3}{k_4}\frac{\Upsilon_{Cl^-}^3}{\Upsilon_{ZnCl_4^{2-}}}\right)} \quad \text{(S6)}$$

$$[Cl^-] = [H^+] + 2[Zn^{2+}] + [ZnCl^+] - [ZnCl_3^-] - 2[ZnCl_4^-] \quad \text{(S7)}$$

For the HCl+$FeCl_2$ system, Eqs. from S8 to S11 were considered for the dissociation species concentrations evaluation:

$$[FeCl^+] = \frac{[Fe^{2+}][Cl^-]}{k_1}\frac{\Upsilon_{Fe^{2+}}\Upsilon_{Cl^-}}{\Upsilon_{FeCl^+}} \quad \text{(S8)}$$

$$[Fe]_{TOT} = [FeCl^+] + [Fe^{2+}] \quad \text{(S9)}$$

$$[Fe^{2+}] = \frac{[Fe]_{TOT}}{1 + \frac{[Cl^-]}{k_1}\frac{\Upsilon_{Fe^{2+}}\Upsilon_{Cl^-}}{\Upsilon_{FeCl^+}}} \quad \text{(S10)}$$

$$[Cl^-] = [H^+] + 2[Fe^{2+}] + [FeCl^+] \tag{S11}$$

The ions activity coefficients reported in Eqs. S1-S11 were evaluated from Davies equation[1] which gives the mean activity coefficient of an electrolyte that dissociates into ions having charges z_1 as a function of ionic strength I:

$$-\log \Upsilon_{\pm} = 0.5042 z_1^2 \left(\frac{\sqrt{I}}{1+\sqrt{I}} - 0.3I \right) \tag{S12}$$

Specifically, for the HCl+$ZnCl_2$ system Eqs. S13 and S14 were considered, while for the HCl+$FeCl_2$ system Eqs. S15 and S16 are reported:

$$\Upsilon_{\pm} \simeq \Upsilon_{Cl^-} \simeq \Upsilon_{ZnCl^+} \simeq \Upsilon_{ZnCl_3^-} \tag{S13}$$

$$\Upsilon_{2\pm} \simeq \Upsilon_{Zn^{2+}} \simeq \Upsilon_{ZnCl_4^{2-}} \tag{S14}$$

$$\Upsilon_{\pm} \simeq \Upsilon_{Cl^-} \simeq \Upsilon_{FeCl^+} \tag{S15}$$

$$\Upsilon_{2\pm} \simeq \Upsilon_{Fe^{2+}} \tag{S16}$$

The dissociation % species is defined as the ratio between the concentration of the different species in solution and the total metal concentration.

$$dissociation\ \%_{species} = \frac{[species]}{[Metal]_{TOT}} \times 100 \tag{S17}$$

It is thus possible to evaluate the dissociation factor by multiply the different dissociation % species by the number of discrete ions obtained from the dissociation reactions of $ZnCl_2$ or $FeCl_2$ that produce the particular species:

$$ZnCl_{2\,dissociation\ factor} = \frac{3[Zn^{2+}] + 2[ZnCl^+] + [ZnCl_2] + [ZnCl_3^-] + [ZnCl_4^{2-}]}{[Zn]_{TOT}} \tag{S18}$$

$$FeCl_{2\,dissociation\ factor} = \frac{3[Fe^{2+}] + 2[FeCl^+]}{[Fe]_{TOT}} \tag{S19}$$

References

1. Davies, C. W. *Ion Association*; Butterworths: London, 1962.
